# Characterization of a Novel Serine Protease Inhibitor Gene from a Marine Metagenome

**DOI:** 10.3390/md9091487

**Published:** 2011-09-05

**Authors:** Cheng-Jian Jiang, Zhen-Yu Hao, Rong Zeng, Pei-Hong Shen, Jun-Fang Li, Bo Wu

**Affiliations:** 1 State Key Laboratory for Conservation and Utilization of Subtropical Agro-bioresources, The Key Laboratory of Ministry of Education for Microbial and Plant Genetic Engineering, and College of Life Science and Technology, Guangxi University, 100 Daxue East Road, Nanning, Guangxi 530004, China; E-Mails: jiangcj0520@gmail.com (C.-J.J.); hzy8517@163.com (Z.-Y.H.); zengrong061@163.com (R.Z.); sos89@126.com (P.-H.S.); ljjf@gxu.edu.cn (J.-F.L.); 2 College of Chemistry and Ecology Engineering, Guangxi University for Nationalities, 188 Daxue East Road, Nanning, Guangxi 530006, China

**Keywords:** serine protease inhibitor, uncultured marine microorganisms, sequenced-based screening, functional characterization

## Abstract

A novel serine protease inhibitor (serpin) gene designated as *Spi1C* was cloned via the sequenced-based screening of a metagenomic library from uncultured marine microorganisms. The gene had an open reading frame of 642 base pairs, and encoded a 214-amino acid polypeptide with a predicted molecular mass of about 28.7 kDa. The deduced amino acid sequence comparison and phylogenetic analysis indicated that Spi1C and some partial proteinase inhibitor I4 serpins were closely related. Functional characterization demonstrated that the recombinant Spi1C protein could inhibit a series of serine proteases. The Spi1C protein exhibited inhibitory activity against α-chymotrypsin and trypsin with *K*_i_ values of around 1.79 × 10^−8^ and 1.52 × 10^−8^ M, respectively. No inhibition activity was exhibited against elastase. Using H-d-Phe-Pip-Arg-pNA as the chromogenic substrate, the optimum pH and temperature of the inhibition activity against trypsin were 7.0–8.0 and 25 °C, respectively. The identification of a novel serpin gene underscores the potential of marine metagenome screening for novel biomolecules.

## 1. Introduction

Serine protease inhibitors (serpins) are the largest and most important superfamily of protease inhibitors. They act as suicide substrates by covalently binding to their target protease, leading to inactivation [1]. Serpins have been found in abundance in eukaryotes, and even in some bacteria as well as archea [2]. Serpins are very interesting because they act as modulators, playing key roles in regulating the activities of numerous serine and cysteine proteases. Serpins control complement activation and a variety of other physiological functions, such as blood coagulation, fibrinolysis, inflammation, tumor cell metastasis, apoptosis, and others [3]. Interestingly, several human serpins and those of other organisms have evolved functions that do not involve protease inhibition [4,5].

The roles of these serpins are being elucidated in animal models of disease. Clearly, many serpins not only inhibit proteases, but also have other physiological effects induced by their interactions with other molecules. These interactions reinforce a particular physiological response [6]. The structure and function of serpins enable them to provide novel scaffolds for engineering protease inhibitors of desired specificity for therapeutic use [7]. Some individual serpins can even efficiently inhibit more than one class of proteases [8]. The structure and properties of serine proteases in covalent complexes are still somewhat uncertain. Studies on the structures of serpin complexes with target proteases are limited, and are mostly focused on complexes with caspases [9,10]. Novel serpin engineering shows considerable potential in creating novel therapeutics that can normalize dysfunctional proteases [11]. The exploration of novel serpins from uncultured microorganisms via genetic biotechnology is interesting. These serpins may be used in candidate antigen gene tests and immunoassays [12].

Metagenomics is a powerful tool for assessing genetic information from uncultured microorganisms. Metagenomics consists of the extraction, cloning, and analysis of an entire genetic material in a given marine habitat [13].

The diversity of marine microbes and their unique environmental properties in extreme conditions potentially contribute to the novelty and value of serpin genes. The serpins encoded by these genes may exhibit unique characteristics that may be useful in specific therapeutic applications [14,15]. To expand the current knowledge on serpins, the present study screened a plasmid metagenomic library [16] constructed from marine water samples. The isolated novel serpin gene was overexpressed in *Escherichia coli*. The results demonstrate the ability of metagenomics to be a powerful tool for discovering novel pharmaceutical enzymes. The potential for marine microbes to become valuable sources of serpins and other industrial enzymes is also proven [17]. To our knowledge, the present study is the first report on a serpin gene isolated from a marine metagenome.

## 2. Results and Discussion

### 2.1. Cloning and Sequence Analysis of a Novel Serpin Gene

Metagenomics strategies are successfully employed to isolate and identify enzymes with novel biocatalytic activities, or secondary metabolites from uncultivable components of microbial communities from various environmental samples [18]. A previous study has constructed a plasmid library containing approximately 50,000 clones from metagenomic DNA isolated from marine water samples [16]. The marine water samples were collected from the South China Sea (21°28′N, 109°07′E). The pH, temperature, and salinity of the collected seawater sample were 8.2, 15 °C, and 32‰, respectively. Plasmid DNA from randomly picked clones is digested with *Eco*RI. Different insert DNAs 1–15 kb long, with an average of approximately 4.0 kb, are obtained. This result confirms that the marine metagenomic library contained DNA molecules from uncharacterized genomes. The marine metagenome of naturally occurring microrganisms is also demonstrated to contain an immense pool of genes. Most of these genes are not represented in pure and enriched cultures established under certain selective conditions [19]. In the present study, a metagenomic library was screened using a sequence-based screening strategy. Consequently, an interesting recombinant clone, named pGXAG59, was identified. pGXAG59 possibly contained a serpin gene, and had its insert further characterized. The insert DNA had a length of 1128 bp, and had no good match with known genes at the DNA level in the database. However, insert DNA was most similar with some serpins at the amino acid level. Based on the database comparison, the cloned gene on pGXAG59 was considered as novel, and was named *Spi1C*. The gene had a long open reading frame of 642 bp, with a mol% G + C content of 49.92, encoding 214 amino acids. The predicted relative molecular weight (Mr) was approximately 24 kDa, and the isoelectric point was 4.33. These values were consistent with the observed Mr of 20–40 kDa of most serpin proteins [20]. Given that the unstable index was 24.90, Spi1C was considered as a relatively stable serpin. The deduced amino acid sequence of Spi1C was searched in the National Center for Biotechnology Information (NCBI) and Expert Protein Analysis System (ExPaSy) databases. The results showed that Spi1C protein shared some moderate similarities with serpins from *Salinibacter ruber* M8 (39% identical and 60% similar) and *Spirosoma linguale* DSM 74 (43% identical and 57% similar). Similarities with three other possible partial proteinase inhibitor I4 serpins were also found. The proteinase inhibitor I4 serpins were from *Dyadobacter fermentans* DSM 18053 (GenBank accession No. YP_003088034; 39% identical and 60% similar), from *Arthrospira maxima* CS-328 (GenBank accession No. ZP_03274562; 38% identical and 59% similar), and from *Cyanothece* sp. PCC 7822 (GenBank accession No. YP_003888593; 37% identical and 58% similar). A similarity with the hypothetical protein BACCELL_01031 (GenBank accession No. ZP_03676704) from *Bacteroides cellulosilyticus* DSM 14838 (36% identical and 60% similar) was also observed.

**Figure 1:**
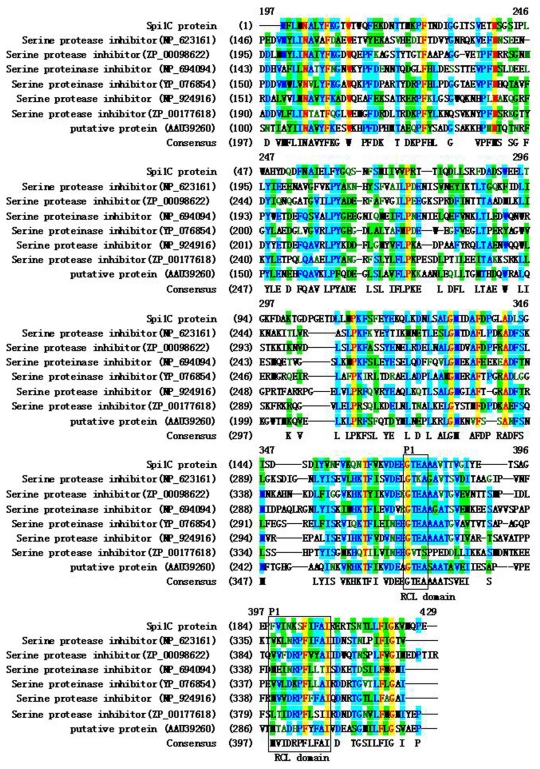
Sequence alignment of Spi1C protein with other bacterial serpins. The serpins are identified by their GenBank accession numbers. The sequence similarity searches were performed using the Basic Local Alignment Search Tool (BLAST) 2.0 program. The amino acid sequence alignment of the target putative protein with homologous proteins was performed using the AlignX program, a component of the Vector NTI suite (InforMax, North Bethesda, MD, USA), using the blosum62mt2 scoring matrix. P1 denotes the start codon of reactive center loop (RCL) conserved domain. The potential RCL sequence regions are in boxed.

Multiple alignments of the deduced amino acids of Spi1C with the most homologous serpin proteins (NCBI database) are shown in Figure 1. The alignments revealed that the target enzymes had lower overall amino acid homologies with other serpins. The percentages of identity with the serpins from bacteria were slightly higher than with those from eukaryotic homologues. The amino acid sequence comparison revealed that the deduced Spi1C peptide shared conserved active site residues with other bacterial serpin members. An amino acid N-terminal extension presented only in the bacterial serpins (Figure 1). Therefore, Spi1C may be classified as a new proteinase inhibitor I4 serpin [21,22].

**Figure 2:**
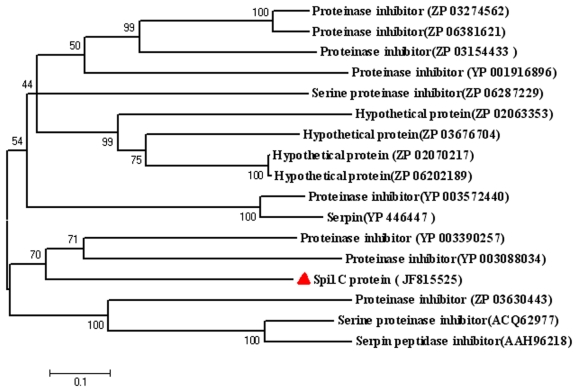
Phylogenetic relationship of the Spi1C protein with other serpins. The sequence alignment was performed using ClustalW version 1.81. The phylogenetic tree was constructed by the neighbor-joining method using Molecular Evolutionary Genetics Analysis (MEGA) version 4.0. Boot-strapping values were used to estimate the reliability of the phylogenetic reconstructions (1000 replicates). The numbers associated with the branches refer to bootstrap values (confidence limits) representing the substitution frequencies per amino acid residue. The serpins are identified by their GenBank accession numbers.

A phylogenetic tree based on the neighbor-joining method located the Spi1C protein in a distinct clade from the serpins found in other microorganisms (Figure 2). Such a placement suggested a relatively high level of divergence from bacterial serpins. The phylogenetic analysis also revealed that the Spi1C protein is not distinctively grouped with the eukaryotic homologues. This finding reflected the considerable dissimilarities between eukaryotes and prokaryotes. Sequence comparisons between the Spi1C protein and other serpins demonstrated differences clustered at the 5′ end of the coding sequence [23].

### 2.2. Overexpression and Purification of Recombinant Spi1C Protein

**Figure 3:**
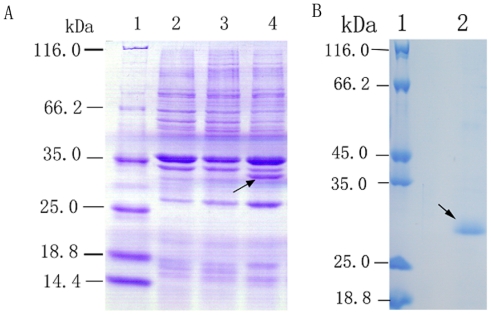
SDS-PAGE of recombinant Spi1C protein. The proteins were separated by 12% (w/v) SDS-PAGE, and then stained with Coomassie brilliant blue G-250. (**A**) Lane 1, molecular weight standards; lane 2, total protein of *E. coli* BL21(DE3)pLysS harboring empty pETBlue-2 as control; lane 3, total protein of *E. coli* BL21(DE3)pLysS harboring the recombinant *spi1C* in pETBlue-2 without induction by isopropyl-β-d-thiogalactopyranoside (IPTG); and lane 4, total protein of *E. coli* BL21(DE3)pLysS harboring the recombinant Spi1C in pETBlue-2 induced by the addition of 0.5 mM IPTG. The recombinant Spi1C protein is indicated by the black arrow; (**B**) Lane 1, molecular weight standards; lane 2, sample purified by the Ni-NTA column method.

To investigate the biochemical properties of Spi1C, the gene was subcloned in a frame with a 6-histidine tag sequence into the expression vector pETBlue-2. The clone was then expressed in *E. coli* BL21(DE3)pLysS. The initial analysis of the crude cell lysate showed that the bacteria containing the recombinant plasmid pETBlue-2-Spi1C produced a substantial amount of the expected recombinant protein. In contrast, this protein was not detectable in the culture of the bacteria containing the parent vector pETBlue-2. The cell extracts of Spi1C were subjected to sodium dodecyl sulfate-polyacrylamide gel electrophoresis (SDS-PAGE). An increased expression of the ~28 kDa protein was observed in the cell extracts of recombinant Spi1C compared with the control. The molecular weights of the proteins were similar with those of recombinant Spi1C. Hence, Spi1C was considered as intracellularly expressed without any modification. The recombinant Spi1C protein was then purified with nickel*-*nitrilotriacetic acid (Ni*-*NTA) metal-affinity chromatography (Figure 3). The purified proteins produced a single band on the SDS-PAGE gels. Their molecular weights concurred with those deduced from the amino acid sequences of the recombinant protein.

### 2.3. Functional Characterization of Recombinant Spi1C Protein

**Figure 4:**
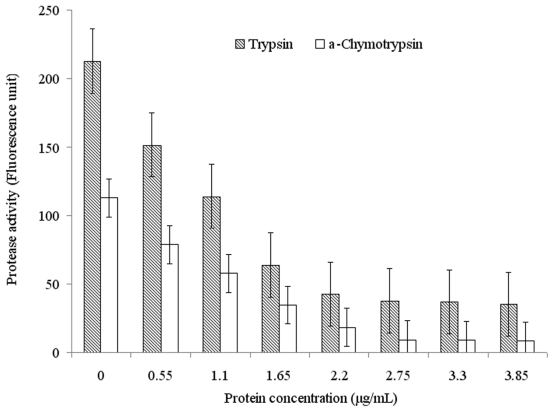
Inhibition effects of the purified recombinant Spi1C protein against trypsin and α-chymotrypsin. Fluorescently labeled trypsin and α-chymotrypsin were incubated with 340 μmol/L of the indicated enzyme at the optimal reaction conditions, with and without the indicated concentration of Spi1C protein, after which the fluorescence was measured.

**Figure 5:**
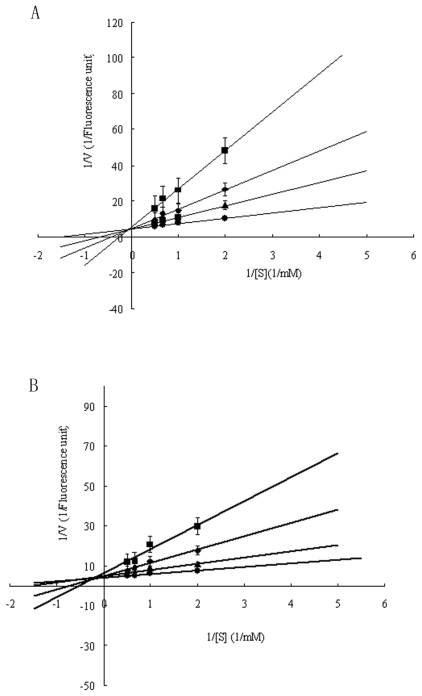
(**A**) Different Lineweaver-Burk plots of the purified recombinant Spi1C protein at different concentrations of trypsin substrate at the optimal reaction conditions; (**B**) Different Lineweaver-Burk plots of the purified recombinant Spi1C protein at different concentrations of α-chymotrypsin substrate with 50 mM Tris-HCl buffer at pH 7.8 and 25 °C. The used different concentrations purified recombinant Spi1C protein were (■) (68.1 nmol/L); (◆) (45.4 nmol/L); (▴) (22.7 nmol/L); and (●) (0 nmol/L), respectively.

**Figure 6:**
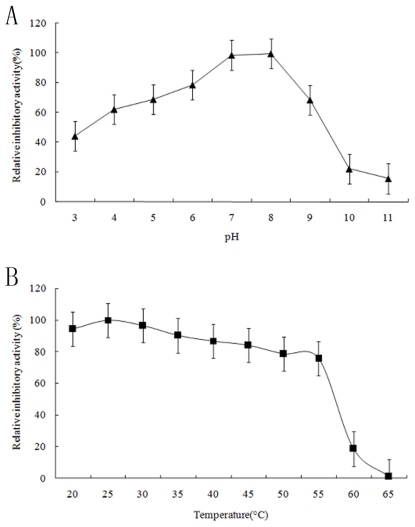
(**A**) Effects of pH (▴) on the inhibitory enzymatic activity of the purified recombinant Spi1C protein; (**B**) Effects of temperature (■) on the inhibitory enzymatic activity of the purified recombinant Spi1C protein. The inhibitory activity was assayed as described in the Experimental Section. The initial trypsin inhibitory activity assayed under standard conditions was considered as 100% relative activity. Values represent the mean ± SD of three independent experiments.

Serpin inhibitors commonly contained two types of inhibition-activity and non-inhibition-activity members. The activity-serpin inhibitors are effective against the serine and cysteine protease families (also including the tissue protease and Cys-Asp families) [1]. The activity-serpin inhibitors (specially the Kunitz and Kazal family members) use reactive site loop structure conformational change and consequent kinetic trapping of an enzyme intermediate to effect inhibition [24]. To examine the inhibitory specificity of the sample to serine proteases, the purified Spi1C protein from a marine metagenome was assayed for inhibitory activity against three different proteases of elastase, trypsin, and α-chymotrypsin (Figure 4). The purified Spi1C protein inhibited trypsin and α-chymotrypsin, but had no effect on elastase. Among the serine proteases, Spi1C protein had inhibitory activity against trypsin, with an inhibitory constant *K*_i_ of 1.52 × 10^−8^ M. However, Spi1C had a comparatively weaker inhibition toward α-chymotrypsin, with an inhibitory constant of 1.79 × 10^−8^ M. Yang *et al*. have found a novel serine protease inhibitor named bicolin from the venom of *Vespa bicolor Fabricius*. The *K*_i_ of the bicolin toward trypsin was 5.5 × 10^−7^ M and no inhibition was detected to elastase and α-chymotrypsin, respectivley [21]. Lu *et al*. have also reported that a novel serine protease inhibitor (Bungaruskuni) from *Bungarus fasciatus* venom had a *K*_i_ of around 6.1 × 10^−6^ M to chymotrypsin [22]. Using different concentrations of Spi1C, the *V*_max_ values of trypsin and α-chymotrypsin remained consistent (Figure 5). These data indicated that Spi1C was a competitive inhibitor. The specific inhibitor activity of Spi1C to trypsin and α-chymotrypsin were 6940 and 3640 U/mg, respectively. Using trypsin as the chromogenic substrate, the optimum pH and temperature of Spi1C against trypsin activity were 7.0–8.0 and 25 °C, respectively (Figure 6). In 2009, Torres-Castillo *et al.* have found that the serpin OsTI 2 from the seeds of *Opuntia streptacantha* only inhibits the trypsin-like proteinases present in *P. truncatus*, *P. americana*, *Acheta* sp., and *Gryllus* sp. [20].

Serpins are easily identified by conserved amino acid motifs because of their high degree of structural conservation. The relationship between the function and structure of the Spi1C protein is interesting. Many kinds of serpins have been separated and characterized from living organisms using culture-dependent techniques [1]. In the current study, a novel serpin gene was identified from a marine metagenome library. However, some serpins may not have inhibitory activity. In the hinge region in the RCL conserved domain, the residues are large and polar (e.g., Lys, Asp, Glu, and Ser); in inhibitory serpins, this region is occupied by small residues (e.g., Ala, Thr, and Ser). Laskowski and Kato [23] have pointed out that P1 site residues of RSL conserved domain in trypsin inhibitors are positively charged residues (Arg or Lys). In the chymotrypsin inhibitor, the residue are usually large and hydrophobic (Leu, Phe, or Tyr). The P1-reactive-site residue of the venom basic protease inhibitor IX has been identified as Asn17, whereas the same position in 2 RCLs of Spi1C is possible Gly and Phe, respectively (Figure 1). Recently, the P1 site residues His and Asn for α-chymotrypsin binding have also been reported [10]. The specificity of Kunitz/bovine pancreatic trypsin inhibitors toward serine proteases is closely associated with P1 amino acids. However, aside from surrounding the reactive site, the residues present in the weak contact loop are also important in different interactions with various serine proteases [23,25]. Thr and Leu were often at the P1 and P1′ positions of RCL, and the protein contained most of the conserved residues [26,27]. Apart from the strange RCL sequence, Spi1C had the inhibitory characteristics of trypsin and α-chymotrypsin. The functional characterization of Spi1C may provide new insights into the relationship among the sequence, structure, and activity of known serpins [27]. Elucidating the catalytic mechanism via studies of the three-dimensional structures of the Spi1C protein using X-ray diffraction methods is an interesting research direction.

## 3. Experimental Section

### 3.1. Plasmid Vectors, Bacterium Strains, and Growth Conditions

The protein expression vector used was pETBlue-2. *E. coli* NovaBlue (Novagen) strain was used as the screening host for the pETBlue-2 bearing inserts. The bacterial strain for the expression of the recombinant protein was *E. coli* BL21(DE3)pLysS (Novagen). All *E. coli* bacterial cultures were grown at 37 °C on Luria–Bertani (LB) agar plates or media. Where appropriate, the media were modified with 100 μg ampicillin/mL, 50 μg carbenicillin/mL, 15 μg tetracycline/mL, or 34 μg chloramphenicol/mL.

### 3.2. DNA Manipulation and Protein Analysis

All DNA manipulations, including cloning and subcloning, transformation of *E. coli* cells, and polymerase chain reaction (PCR), were performed according to standard techniques [28] or following the instructions of the manufacturer, unless indicated otherwise. Protein preparation and analysis, including protein extraction from *E. coli*, protein quantification, and SDS-PAGE, were performed as described by standard protocols [29].

### 3.3. DNA Sequence Analysis, Database Search, and Gene Structure Characterization

DNA sequence analysis was performed using the BigDye Terminator Cycle sequencing kit on an ABI Prism 3700 DNA analyzer (Applied Biosystems, USA). Protein translation was carried out using the web-based translation tool at the ExPaSy homepage [30]. Sequence predictions were retrieved from the protein and nucleotide databases at NCBI Entrez page [31]. Sequence similarity searches were performed with the BLAST 2.0 program. The amino acid sequence alignment of Spi1C with homologous proteins was done using the AlignX program, a component of the Vector NT1 suite (InforMax, North Bethesda, MD, USA) using the blosum62mt2 scoring matrix. Based on the comparison of the deduced amino acids, a putative gene (*Spi1C*) encoding a novel serpin was identified and further characterized. A phylogenetic tree was constructed using the neighbor-joining method with MEGA 4.0 software [32]. Boot-strapping values were used to estimate the reliability of the phylogenetic reconstructions (1000 replicates).

### 3.4. Overexpression and Purification of the Recombinant Serpin Protein

The *Spi1C* nucleotide sequence was amplified from the plasmid pGXAG59 isolated from a serpin-producing clone. PCR was carried out in a total volume of 50 μL containing 2.5 mM MgCl_2_, 10 mM Tris-HCl (pH 8.4), 50 mM KCl, 0.2 mM deoxynucleotide triphosphate, 0.4 μM each primer, 1.0 unit Vent DNA polymerase (NEB, USA), and 10 ng of plasmid template. The restriction enzyme sites (underlined) for *Bam*HI and *Hin*dIII were designed in the forward primer/reverse primer (5′-TTAGGATCCGATGTTCCTTATGAACGCC-3′/5′-ATAAGCTTCTCCGGCTGCATCACTTTC-3′). The PCR cycling consisted of denaturation steps (96 °C for 2 min), 30 cycles at 94 °C for 40 s, 58.5 °C for 30 s, and 72 °C for 2 min, as well as a final extension step at 72 °C for 10 min. After amplification, the PCR product mixture was digested by *Bam*HI as well as *Hin*dIII, and directly ligated into the pETBlue-2 (Novagen) expression vector cleaved with the same enzymes. The resulting recombinant plasmids were transferred into NovaBlue (Novagen) competent cells and placed on LB selection plates. After overnight incubation at 37 °C, positive white colonies were picked for the isolation of the recombinant expression plasmid. The recombinant expression plasmid was then introduced into *E. coli* BL21(DE3)pLysS to express the target protein.

The transformed bacterial cells were cultured in an LB medium containing 50 μg carbenicillin/mL and 100 μg chloramphenicol/mL at 37 °C. Protein expression was induced by the addition of 1 mM isopropyl-β-d-thiogalactopyranoside when the optical density at 600 nm reached 0.6. After incubation for an additional 6 h, the cells were harvested, washed twice with phosphate-buffered saline (pH 7.6), and lysed by sonication in 10 mL of 20 mM Tris-HCl (pH 8.0). The lysate was centrifuged twice at 30,000× *g* for 20 min at 4 °C. About 1 mL of the supernatant was diluted with 5 mL of column buffer (20 mM Tris-HCl, pH 8.0, 10 mM imidazole, 300 mM NaCl). The diluted supernatant was then applied onto an equilibrated Ni-NTA column containing 1 mL of Ni-NTA-agarose (Novagen). After washing with 5 mL of column buffer containing 10 mM imidazole, the recombinant protein was eluted with column buffer containing 250 mM imidazole and the target fraction (~600 μL) was collected. Immediately after elution from the column, 1,4-dithiothreitol (Promega) was added to the protein solution to a final concentration of 5 mM. The protein concentration was determined using a Bio-Rad protein assay kit, with bovine serum albumin as the standard. The protein purified with the Ni-NTA column was used for enzyme activity assays.

### 3.5. Characterization of the Serpin Gene *Spi1C*

#### 3.5.1. Elastase Inhibition Assay

The inhibition effects of *Spi1C* against the hydrolysis of the synthetic chromogenic substrate elastase (final concentration 20 nM) were assayed in 50 mM Tris-HCl (pH 7.8) at 30 °C. The final concentration, approximately 0.5 μM of inhibitor, was pre-incubated for 10 min at 30 °C. *N*-benzoyl-Arg-4-nitroanilide-hydrochloride-p-nitroaniline (pNA) (Sigma) was used as a substrate of elastase [33]. The reaction was initiated by the addition of the substrate to a final concentration of 0.5 mM. The formation of pNA was continuously monitored at 405 nm for 5 min. The effect of the inhibitor was estimated by setting the initial velocity obtained in the presence of the enzyme alone (without inhibitor) as 100%. The inhibition assay was carried out per above process, and *K*_i_ was obtained by the reciprocal plotting of the reaction chromogenic substrate concentrations (0–2 mM).

#### 3.5.2. α-Chymotrypsin Inhibition Assay

α-Chymotrypsin activity was determined by an adapted method reported by Kang and Fuchs [34] with slight modifications. The blank was prepared with a buffer (50 mM Tris-HCl, pH 7.8 at 25 °C), and 0.5 mM *N*-benzoyl-tyrosine ethyl ester (BTEE; Sigma) was used as the substrate. The reaction was continuously monitored at 253 nm for 5 min. The effect of the inhibitor was estimated by setting the initial velocity as 100%, obtaining the enzyme alone (without the Spi1C protein). The inhibition assay was carried out as described above. *K*_i_ was obtained by the reciprocal plotting of the reaction velocity via inhibitor concentration under different substrate concentrations (0–2 mM). A control enzyme was determined using a buffer solution (990 μL) with 10 μL of enzyme (1 mg/mL), and incubating with 2 mL of BTEE for 3 min. Inhibitory activity was carried out for 3 min at 25 °C, and the residual enzymatic activity was measured.

#### 3.5.3. Trypsin Inhibition Assay

The multi-reaction assay for trypsin inhibitory activity was based on H-d-Phe-Pip-Arg-pNA (S-2238; Kabi Vitrum, Stockholm, Sweden) hydrolysis according to Elanger *et al*. [35] with slightly modifications. One trypsin inhibitory activity unit was defined as the decrease in 0.01 unit of absorbance per 15 min at 25 °C. The reaction was initiated by the addition of the substrate to a final concentration of 0.5 mM. The formation of pNA was continuously monitored at 405 mM for 5 min. The blank was prepared with 50 mM Tris-HCl buffer (pH 7.8) and 0.5 mM S-2238 substrate at 25 °C. The enzyme-controlled reaction was investigated under the same conditions with the addition of S-2238 (0.5 mM) in deionized H_2_O incubated for 15 min. The inhibitory activity was determined using an aliquot of the enzyme similar with the control reaction with 0.5 μM inhibitor extract. This mixture was pre-incubated for 15 min to allow the formation of the proteinase-inhibitor complex before substrate addition. Upon substrate addition, hydrolysis was allowed to proceed for 15 min, and the remaining activity was measured. The profiles of activity *versus* pH and activity *versus* temperature were determined using the above standard assay method. All experiments were performed in triplicate. To determine the effect of pH on the inhibitory activity of the recombinant Spi1C protein, the inhibitor activity was assayed within pH 3.0–8.0 in 50 mM citric acid-sodium citrate buffer, with 50 mM Tris-HCl (pH 7.0–8.5) and 100 mM glycine-NaOH (pH 8.5–11.0) buffers under standard conditions. To assess the effect of temperature on Spi1C activity, the inhibitor was assayed at various temperatures (20–100 °C) in 50 mM Tris-HCl buffer (pH 8.0). The inhibition assay was carried out as described above, and *K*_i_ was obtained by the reciprocal plotting of the reaction velocity via inhibitor concentration under different substrate concentrations (0–2 mM).

### 3.6. Nucleotide Sequence Accession Number

The *Spi1C* nucleotide sequence was deposited in GenBank under accession number **JF815525**.

## 4. Conclusions

A novel serpin gene, *Spi1C*, was identified by the sequence-based screening of a metagenomic library from uncultured marine microorganisms. Sequence analysis suggested that the identified gene product was related to proteinase inhibitor I4 serpins. Functional characterization demonstrated that the recombinant Spi1C protein exhibited comparable inhibitory activity with trypsin and α-chymotrypsin. More detailed biochemical characterizations of Spi1C are currently in progress. Identifying a new serpin from marine microorganisms emphasizes the advantages of a metagenomic library in cloning novel genes via sequence-based screening using *E. coli* hosts [27,36].

## References

[1] Gettins P (2002). Serpin structure, mechanism, and function. Chem. Rev.

[2] Silverman GA, Whisstock JC, Bottomley SP, Huntington JA, Kaiserman D, Luke CJ, Pak SC, Reichhart JM, Bird PI (2010). Serpins flex their muscle: Putting the clamps on proteolysis in diverse biological systems. J. Biol. Chem.

[3] Gooptu B, Lomas DA (2009). Conformational pathology of the serpins: Themes, variations, and therapeutic strategies. Annu. Rev. Biochem.

[4] Belorgey D, Hägglöf P, Karlsson-Li S, Lomas DA (2007). Protein misfolding and the serpinopathies. Prion.

[5] Gettins PGW, Olson ST (2009). Exosite determinants of serpin specificity. J. Biol. Chem.

[6] Davis AE, Mejia P, Lu F (2008). Biological activities of C1 inhibitor. Mol. Immunol.

[7] Dementiev A, Dobo J, Gettins PGW (2006). Active-site distortion is sufficient for proteinase inhibition by serpins. J. Biol. Chem.

[8] Quan LT, Caputo A, Bleackley RC, Pickup DJ, Salvesen GS (1995). Granzyme B is inhibited by the cowpox virus serpin cytokine response modifier A. J. Biol. Chem.

[9] Schick C, Pemberton PA, Shi GP, Kamachi Y, Cataltepe S, Bartuski AJ, Gornstein ER, Brömme D, Chapman HA, Silverman GA (1998). Cross-class inhibition of the cysteine proteinases cathepsins K, L, and S by the serpin squamous cell carcinoma antigen 1: A kinetic analysis. Biochemistry.

[10] Scheidig AJ, Hynes TR, Pelletier LA, Wells JA, Kossiakoff AA (1997). Crystal structures of bovine chymotrypsin and trypsin complexed to the inhibitor domain of Alzheimer’s amyloid beta-protein precursor (APPI) and basic pancreatic trypsin inhibitor (BPTI): Engineering of inhibitors with altered specificities. Protein Sci.

[11] Nagahara A, Nakayama M, Oka D, Tsuchiya M, Kawashima A, Mukai M, Nakai Y, Takayama H, Nishimura K, Jo Y, Nagai A, Okuyama A, Nonomura N (2010). SERPINE2 is a possible candidate promotor for lymph node metastasis in testicular cancer. Biochem. Biophys. Res. Commun.

[12] Kennedy J, Flemer B, Jackson SA, Lejon DP, Morrissey JP, O’Gara F, Dobson AD (2010). Marine metagenomics: New tools for the study and exploitation of marine microbial metabolism. Mar. Drugs.

[13] Newton RJ, Griffin LE, Bowles KM, Meile C, Gifford S, Givens CE, Howard EC, King E, Oakley CA, Reisch CR, Rinta-Kanto JM, Sharma S, Sun S, Varaljay V, Vila-Costa M, Westrich JR, Moran MA (2010). Genome characteristics of a generalist marine bacterial lineage. ISME J.

[14] Johnson DJ, Langdown J, Huntington JA (2010). Molecular basis of factor IXa recognition by heparin-activated antithrombin revealed by a 1.7-A structure of the ternary complex. Proc. Natl. Acad. Sci. USA.

[15] Jean F, Stella K, Thomas L, Liu G, Xiang Y, Thomas G (1998). alpha1-Antitrypsin Protland, a bioengineered serpin highly selective for furin: Application as an antipathogenic agent. Proc. Natl. Acad. Sci. USA.

[16] Jiang C, Wu LL, Zhao GC, Shen PH, Jin K, Hao ZY, Li SX, Ma GF, Luo FF, Hu GQ, Kang WL, Bi YL, Qin XM, Tang XL, Wu B (2010). Identification and characterization of a novel fumarase gene by metagenome expression cloning from marine microorganisms. Microb. Cell Fact.

[17] Yooseph S, Nealson KH, Rusch DB, McCrow JP, Dupont CL, Kim M, Johnson J, Montgomery R, Ferriera S, Beeson K, Williamson SJ, Tovchigrechko A, Allen AE, Zeigler LA, Sutton G, Eisenstadt E, Rogers YH, Friedman R, Frazier M, Venter JC (2010). Genomic and functional adaptation in surface ocean planktonic prokaryotes. Nature.

[18] Singh BK (2010). Exploring microbial diversity for biotechnology: The way forward. Trends Biotechnol.

[19] Edwards JL, Smith DL, Connolly J, McDonald JE, Cox MJ, Joint I, Edwards C, McCarthy AJ (2010). Identification of carbohydrate metabolism genes in the metagenome of a marine biofilm community shown to be dominated by gammaproteobacteria and bacteroidetes. Genes.

[20] Torres-Castillo JA, Jacobo CM, Blanco-Labra A (2009). Characterization of a highly stable trypsin-like proteinase inhibitor from the seeds of *Opuntia streptacantha* (*O. streptacantha* Lemaire). Phytochemistry.

[21] Yang X, Wang Y, Lu Z, Zhai L, Jiang J, Liu J, Yu H (2009). A novel serine protease inhibitor from the venom of *Vespa bicolor* Fabricius. Comp. Biochem. Physiol. B Biochem. Mol. Biol.

[22] Lu J, Yang H, Yu H, Gao W, Lai R, Liu J, Liang X (2008). A novel serine protease inhibitor from *Bungarus fasciatus* venom. Peptides.

[23] Laskowski M, Kato I (1980). Protein inhibitors of proteinases. Annu. Rev. Biochem.

[24] Bode W, Huber R (1992). Natural protein proteinase inhibitors and their interaction with proteinases. Eur. J. Biochem.

[25] Cheng YC, Yan FJ, Chang LS (2005). Taiwan cobra chymotrypsin inhibitor: Cloning, functional expression and gene organization. Biochim. Biophys. Acta.

[26] Irving JA, Pike RN, Lesk AM, Whisstock JC (2000). Phylogeny of the serpin superfamily: Implications of patterns of amino acid conservation for structure and function. Genome Res.

[27] Olson ST, Gettins PG (2011). Regulation of proteases by protein inhibitors of the serpin superfamily. Prog. Mol. Biol. Transl. Sci.

[28] Sambrook J, Russell DW (2001). Molecular Cloning: A Laboratory Manual.

[29] Schägger H, von Jagow G (1987). Tricine-sodium dodecyl sulfate-polyacrylamide gel electrophoresis for the separation of proteins in the range from 1 to 100 kDa. Anal. Biochem.

[30] ExPASy Translate.

[31] NCBI Entres.

[32] Tamura K, Dudley J, Nei M, Kumar S (2007). MEGA4: Molecular Evolutionary Genetics Analysis (MEGA) software version 4.0. Mol. Biol. Evol.

[33] Barrett AJ (1972). A new assay for cathepsin B1 and other thiol proteinases. Anal. Biochem.

[34] Kang SH, Fuchs MS (1973). An improvement in the Hummel chymotrypsin assay. Anal. Biochem.

[35] Ahsan MN, Watabe S (2001). Kinetic and structural properties of two isoforms of trypsin isolated from the viscera of Japanese anchovy, *Engraulis japonicus*. J. Protein Chem.

[36] Kristiansson E, Hugenholtz P, Dalevi D (2009). ShotgunFunctionalizeR: An R-package for functional comparison of metagenomes. Bioinformatics.

